# A Natural Language Processing–Assisted Extraction System for Gleason Scores: Development and Usability Study

**DOI:** 10.2196/27970

**Published:** 2021-07-02

**Authors:** Shun Yu, Anh Le, Emily Feld, Emily Schriver, Peter Gabriel, Abigail Doucette, Vivek Narayan, Michael Feldman, Lauren Schwartz, Kara Maxwell, Danielle Mowery

**Affiliations:** 1 University of Pennsylvania Health System Philadelphia, PA United States; 2 Perelman School of Medicine Philadelphia, PA United States

**Keywords:** NLP, Gleason score, prostate cancer, natural language processing

## Abstract

**Background:**

Natural language processing (NLP) offers significantly faster variable extraction compared to traditional human extraction but cannot interpret complicated notes as well as humans can. Thus, we hypothesized that an “NLP-assisted” extraction system, which uses humans for complicated notes and NLP for uncomplicated notes, could produce faster extraction without compromising accuracy.

**Objective:**

The aim of this study was to develop and pilot an NLP-assisted extraction system to leverage the strengths of both human and NLP extraction of prostate cancer Gleason scores.

**Methods:**

We collected all available clinical and pathology notes for prostate cancer patients in an unselected academic biobank cohort. We developed an NLP system to extract prostate cancer Gleason scores from both clinical and pathology notes. Next, we designed and implemented the NLP-assisted extraction system algorithm to categorize notes into “uncomplicated” and “complicated” notes. Uncomplicated notes were assigned to NLP extraction and complicated notes were assigned to human extraction. We randomly reviewed 200 patients to assess the accuracy and speed of our NLP-assisted extraction system and compared it to NLP extraction alone and human extraction alone.

**Results:**

Of the 2051 patients in our cohort, the NLP system extracted a prostate surgery Gleason score from 1147 (55.92%) patients and a prostate biopsy Gleason score from 1624 (79.18%) patients. Our NLP-assisted extraction system had an overall accuracy rate of 98.7%, which was similar to the accuracy of human extraction alone (97.5%; *P*=.17) and significantly higher than the accuracy of NLP extraction alone (95.3%; *P*<.001). Moreover, our NLP-assisted extraction system reduced the workload of human extractors by approximately 95%, resulting in an average extraction time of 12.7 seconds per patient (vs 256.1 seconds per patient for human extraction alone).

**Conclusions:**

We demonstrated that an NLP-assisted extraction system was able to achieve much faster Gleason score extraction compared to traditional human extraction without sacrificing accuracy.

## Introduction

In recent years, the widespread adoption of electronic health record (EHR) systems has led to a dramatic rise in the amount of clinical data available for research and improvement of patient care. Unfortunately, large amounts of clinical data are found only within medical notes (ie, clinical or pathology notes written by health care providers) and are stored as unstructured free text. Thus, clinically important data require manual extraction by human experts, a process which can be slow, expensive, difficult to scale and reproduce, and prone to human errors.

Natural language processing (NLP), a technology at the intersection of computational linguistics, computer science, and artificial intelligence, can permit much faster and more scalable information extraction compared to manual, human extraction [1]. However, NLP systems typically have difficulty interpreting and extracting information documented within highly complex notes or sentence structures. Although the majority of real-world medical notes provide simple yet accurate clinical information, there is inevitably a proportion of medical notes which can be hard to interpret for NLP systems for a variety of reasons (inaccurate documentation, conflicting information, insufficient context, etc). In theory, an NLP system can not only provide extraction capabilities, but may also distinguish whether the note being extracted is “uncomplicated,” defined as any note easily processed by NLP, or “complicated,” defined as any note not easily processed by NLP. Thus, if during its processing, NLP can successfully discern uncomplicated versus complicated notes, an NLP-assisted extraction system can be devised where uncomplicated notes are allocated for NLP review, while complicated notes are allocated for human review. In essence, the NLP system “assists” the human extractor by reducing his or her workload but does not replace the human entirely. This system leverages the fact that NLP can review and process uncomplicated notes much faster than can humans, while humans are much more accurate than are NLP systems at interpreting and deciphering complicated notes.

We developed and piloted our NLP-assisted extraction system for the collection of prostate cancer Gleason score (GS) data in order to clinically annotate an institutional prostate cancer biobank. GS describes the histologic grade of prostate cancers and plays a crucial role in the prognostication and risk stratification of newly diagnosed prostate cancer patients [2-4]. However, GS is often unavailable in research databases because it is stored as unstructured data within clinical and pathology notes, which require human extraction. There is currently a paucity of NLP solutions for extracting GS from both clinical and pathology notes, and these existing options are limited by either accuracy or scope [5-8].

Thus, we developed an NLP-assisted extraction system for encoding GS from medical notes. We assessed the accuracy and speed of our NLP-assisted extraction system and compared it to extraction with NLP alone and humans alone. We hypothesized that our NLP-assisted extraction system would greatly improve the speed of data extraction compared to human extraction alone, while maintaining the accuracy of human extractors.

## Methods

### Data Ascertainment

For this University of Pennsylvania Institutional Review Board–approved study, we queried all eligible Penn Medicine Biobank patients who were diagnosed with prostate cancer using a combination of International Classification of Disease (ICD) 9 and 10 codes and data from our institution’s cancer registry. This cohort has undergone manual review and represents a reference standard cohort of unselected biobank participants with a current or past history of prostate cancer. Our EHR data is warehoused on the University of Pennsylvania Health System’s EPIC Clarity system. The Penn Data Analytics Center queried this system for all available clinical and pathology notes for each patient in our cohort ranging from January 1, 2001, through January 31, 2020. Clinical notes were defined as any free-text note written by a health care provider in the EHR, including but not limited to office visit notes and telephone notes written by medical oncologists, radiation oncologists, urologists, physician assistants, and nurses. Pathology notes were defined as any free-text note written by the pathology department and associated with a pathology evaluation. Both clinical and pathology notes were collected because GS may be found in both note types.

### Gleason Score Extraction

Our objective was to extract the highest GS from both prostate surgeries and prostate needle biopsies because the highest GS is used clinically for treatment decisions and prognostication. The GS identified from prostate surgery and biopsy may differ, as they are typically obtained at different times and possibly from different areas of the prostate. A fully specified GS comprises 3 components: primary GS (P), secondary GS (S), and total GS (T). As per the International Society of Urological Pathology consensus [9], the primary GS (range 3-5) and secondary GS (range 3-5) describe the first and second most prevalent histology grades in a prostate cancer specimen, respectively. For example, if a prostate biopsy specimen contained 15% grade 3, 55% grade 4, and 30% grade 5, then the primary GS would be 4 and the secondary GS would be 5. The total GS (range 6-10) is defined as the additive sum of the primary and secondary GS (ie, P+S=T). A total GS lower than 6 is possible but is considered benign and not “cancer” and thus did not appear in our prostate cancer cohort.

**Figure 1:**
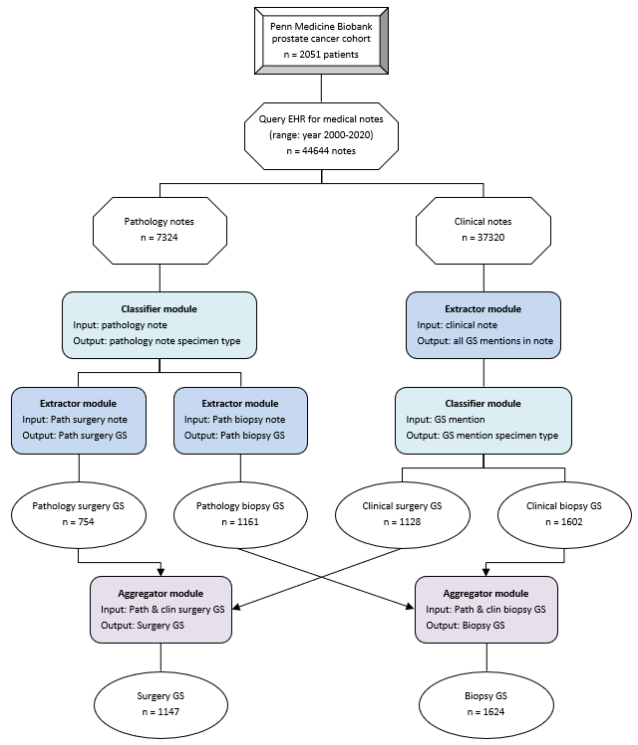
Flow diagram for our Gleason score NLP extractor. EHR: electronic health record.

We developed an NLP system to extract GS from both prostate biopsy and surgery for all patients in our cohort. The NLP extraction process was accomplished using multiple modules in conjunction with each other (Figure 1). We designed 3 types of modules: extractor modules, classifier modules, and aggregator modules. Extractor modules identify mentions of GS in the notes based on a specified lexicon (eg, “GS,” “Gleason,” etc) and then extract the adjacent GS components for each mention of GS. Classifier modules determine whether the extracted GS was derived from a prostate biopsy or prostate surgery using another specified lexicon (eg, “RRP,” indicating retropubic radical prostatectomy, or “PNBx,” indicating prostate needle biopsy). Lexicons were built based on input from clinical experts.

**Figure 2:**
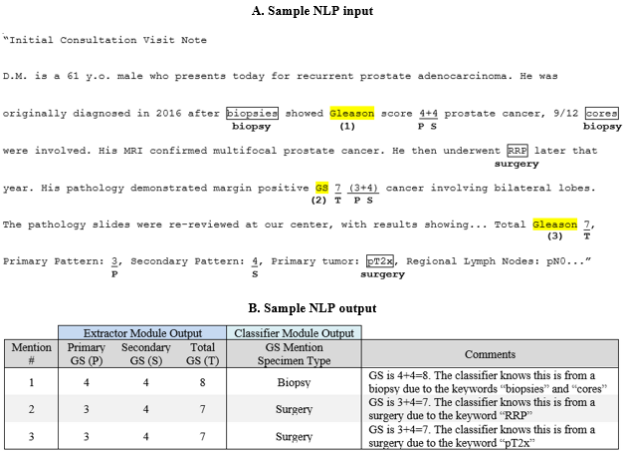
Example of Gleason score extractor and classifier module logic for clinical notes. NLP: natural language processing.

An example of how the extractor and classifier modules work for clinical notes is described in Figure 2. First, the extractor module identifies all mentions of GS (labeled 1, 2, 3, etc) in the clinical note. For each GS found, the extractor module searches the surrounding text for the 3 GS components and then outputs these score components. If only 2 of the 3 components are found, then the third one is derived based on the following equation: P+S=T (eg, T=7 is derived from P=4 and S=3). If no GS is found, the output is documented this way. Second, the classifier module searches the surrounding text, applying a lexicon based on a tiered-priority system where more specific terms (eg “Prostatectomy”) take precedence over less specific terms (eg, “Pathologic Stage”). Furthermore, if no classification is possible based on the initial search, then the search area is broadened. After searching, the classifier module outputs the specimen type of the GS mention: either prostate biopsy or prostate surgery. More details on these algorithms can be found in the simplified pseudocodes in Multimedia Appendix 1 Table S1 A and B.

Finally, the aggregator module calculates and assigns a set of patient-level GS—1 for prostate surgery and 1 for prostate biopsy—for each patient. The final prostate surgery GS is calculated based on the maximum extracted GS from either pathology or clinical notes for any prostate surgery according to the algorithm found in Multimedia Appendix 1 Table S1. Similarly, the final prostate biopsy GS is calculated based on the maximum extracted GS for any prostate biopsy, from either pathology or clinical notes. These final values are subsequently encoded into a structured data format.

Each module was designed from the ground up by a practicing oncologist, SY, so that the NLP system logic would best mirror the mental extraction process performed by clinicians when they are looking for GS in medical notes. For example, due to innate differences in the way GS is typically recorded in clinical versus pathology notes, the extractor and classifier modules were slightly different for the two note types and also arranged differently (see Figure 1 and Table S1 A and B, Multimedia Appendix 1). For pathology notes, a classifier module was applied first, followed by the extractor module. This was because each pathology could only contain either prostate biopsy or surgery information but not both. For clinical notes, the extractor module was applied first followed by the classifier module. This was because each clinical note could have multiple GS mentions, and each of those mentions could be from a different specimen source. Thus, the classifier module could be applied only after the extractor module found a GS mention.

### NLP-Assisted Extraction System

We additionally constructed an NLP algorithm that could distinguish uncomplicated versus complicated notes. The algorithm designates a note as complicated if the extracted information is inaccurate (eg, “Gleason score was 3+4=8”), incomplete (eg, “Primary GS was 4,” but no information was provided on secondary or total GS), or conflicting (eg, prostate surgery GS from pathology note was “4+4=8,” but the clinical note was “4+3=7”; see Table S1 C, Multimedia Appendix 1 for more details). Rather than using objective measures of complexity, we chose to use this set of criteria because it was clinically based and deemed to be a suitable proxy for the level of complexity in the extracted note. Uncomplicated notes were defined as any note not designated as complicated.

### Accuracy Assessment

We randomly selected 200 patients for manual human review to assess the accuracy of our NLP system (100 charts reviewed by author SY and 100 charts reviewed by author AL). During human extraction, the extractor was blinded to the NLP results. Discrepancies between the NLP system and human extraction were then manually reviewed by consensus and analyzed to determine the cause of the discrepancy. Discrepancies were assigned to be due to either NLP error or human error. The accuracy of the NLP system and human extraction were calculated based on the number of NLP and human errors, respectively. Differences in accuracy were calculated using the Fisher exact test. *P* values ≤.05 were considered statistically significant.

## Results

### General Trends

We identified 2051 prostate cancer patients from the Penn Medicine Biobank cohort, for whom 7324 pathology notes and 37,320 clinical notes were queried from our EHR data warehouse. Of note, each patient could have multiple pathology and clinical notes in the EHR (average of 3.6 pathology and 18.2 clinical notes per patient).

**Figure 3:**
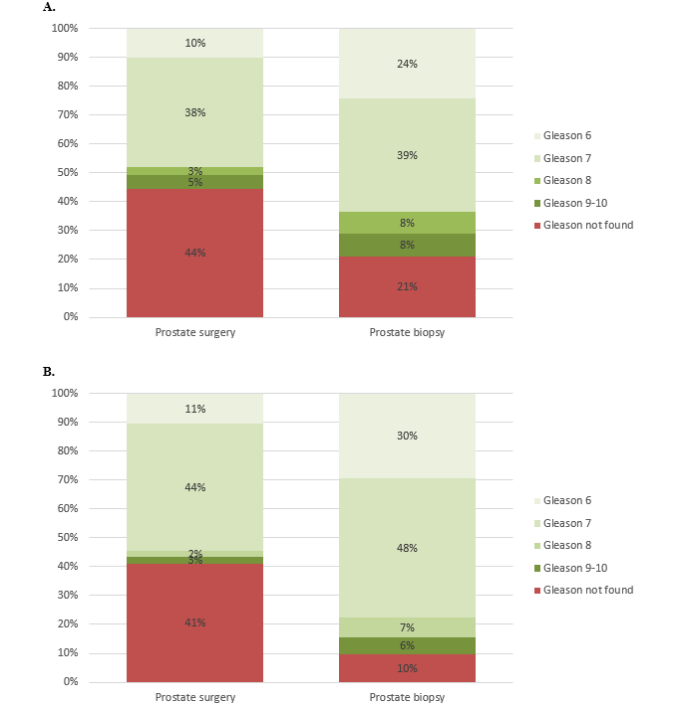
Distribution of NLP Gleason score extractor results for (A) the full cohort and (B) the randomly selected 200 patients. NLP: natural language processing.

Based on the queried pathology and clinical notes, the NLP system successfully produced a result for all 2051 patients in our cohort: either a GS or “not found” if no GS was documented in our EHR. The distribution of results is shown in Figure 3A. The distribution of the prostate surgery GS was higher than that of the prostate biopsy GS as expected, as patients with a lower GS on biopsies are less likely to receive surgery and due to the phenomenon of pathologic upgrading. The NLP system also identified a total of 199/4102 (4.85%) notes as complicated, including 66/2051 (3.23%) prostate surgery and 133/2051 (6.48%) prostate biopsy notes. The remaining notes were therefore identified as uncomplicated.

### Accuracy Assessment

From the full cohort, 200 patients were randomly selected, and a human extractor manually extracted both a prostate surgery and prostate biopsy GS. Thus, a total of 400 GS (200 prostate surgery GS and 200 prostate biopsy GS) was compared to NLP results for accuracy. The distribution of results is shown in Figure 3B.

**Figure 4:**
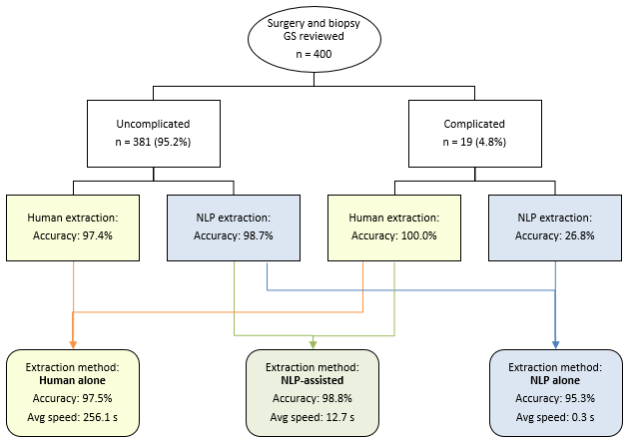
Accuracy and average extraction time for the three extraction methods.

Among these 400 prostate surgeries and biopsies, 19 (4.8%) were identified as complicated (see Figure 4). Among the 381 uncomplicated notes, there were 10 human errors (accuracy 371/381, 97.4%) and 5 NLP errors (accuracy 376/381, 98.7%). Further characterization of the NLP errors is shown in Multimedia Appendix 2 Table S2. Thus, among uncomplicated notes, human and NLP accuracy was similar (*P*=.30). Among the 19 complicated notes, there were 0 human errors (accuracy 19/19, 100.0%) and 14 NLP errors (accuracy 5/19, 26.8%). Thus, among complicated notes, human extraction was significantly more accurate than was NLP extraction (*P*=.02). Details of the breakdown in accuracy between prostate surgeries and biopsies are displayed in Multimedia Appendix 3 Table S3.

With human extraction alone, both uncomplicated and complicated notes would be assigned to human extraction, producing an overall accuracy rate of 97.5%. With NLP extraction alone, both uncomplicated and complicated notes would be assigned to NLP extraction, producing an overall accuracy rate of 95.3%. In the NLP-assisted extraction system, uncomplicated notes were assigned to NLP extraction while complicated notes are assigned to human extraction. This produced an overall accuracy of 98.8%. The overall accuracy of the NLP-assisted extraction system was similar to that of human extraction alone (*P*=.17), while it was significantly more accurate than that of NLP extraction alone (*P*<.001).

### Extraction Time Analysis

The NLP system extracted GS from all of the pathology notes in approximately 60 seconds and from all clinical notes in 486 seconds. In total, the NLP system processed the full data set for 2051 patients in 546 seconds, which equates to approximately 0.27 seconds per patient (see Figure 4). In comparison, human extraction times were much longer: SY required an average of 306.0 seconds per patient, and AL required an average of 206.3 seconds per note. Thus, the average human extraction time was 256.1 seconds per note. In the NLP-assisted extraction model, approximately 5% of notes required human extraction. Thus, the NLP-assisted approach took an estimated weighted average of 12.7 seconds per note.

## Discussion

We constructed an NLP GS extraction system which collected GS from both pathology and clinical notes with high accuracy. We also implemented and assessed an NLP-assisted extraction system that exhibited superior extraction times compared to that of human extraction alone, while maintaining comparable accuracy. Thus, we demonstrated that an NLP-assisted extraction system is capable of using both NLP and human extraction to maximize the strengths of each while overcoming their respective weaknesses.

Charles Friedman [10] states that any health care technology, including NLP systems, should follow the “fundamental theorem” of biomedical informatics: a person working in partnership with an information resource is “better” than that same person unassisted. Our NLP-assisted extraction model aims to achieve this principle by designing our NLP tool to serve as an “intelligent assistant” to the human extractor, working together with humans to create an extraction system which is both fast and accurate.

We believe that this combination of superior accuracy and faster extraction time can greatly accelerate data collection during the establishment of large clinical data warehouses, which can in turn expedite clinical research, quality improvement projects, clinical decision support tools, etc. Although our NLP-assisted extraction model requires approximately 5% of the notes to still be manually reviewed by a human extractor, this also means we can reduce the workload of human extractors by approximately 95%. By extension, this can potentially reduce the cost of variable extraction by approximately 95%, which is important since human extractors represent a major source of cost for establishing most large clinical databases.

NLP solutions have additional benefits. First, NLP can produce highly reliable and standardized extractions compared to human extraction. Extraction style and criteria may vary slightly between different extractors and sometimes even between different times for the same extractor. NLP systems, on the other hand, provide standardized and reproducible results. Second, NLP systems can reduce omission errors due to cognitive biases to which all human tasks are prone. Third, NLP systems are scalable. If a researcher wanted to double the scope of their database, it would require a doubling of the workload for human extractors. However, NLP systems require only electricity and computing costs to execute and therefore can be expanded at scale to meet the needs of researchers with minimal cost.

Finally, the increased speed and scalability of the NLP extraction unlocks important database features that traditional databases lack. For example, NLP extraction allows continuous updates for the database. Because new data are entered by health care workers into the EHR on a daily basis, any clinical database that strives to provide up-to-date clinical data will require human extractors to continuously review new data as they are entered. This task can be both expensive and time-consuming. For example, most current large clinical databases take months to years to provide up-to-date data due the time required for manual human extractors. This limits the ability of clinical researchers and quality-improvement researchers to answer clinically important questions in a timely manner.

Of course, our NLP-assisted model still requires the availability of human extractors, which might not be accessible to all research groups. For researchers hoping to build a clinical database without any human extraction, our NLP system might still provide utility, as it was able to extract GS elements with an accuracy of over 95%.

Currently, there are not many publicly available NLP solutions for adequate extraction of GS. Two previous projects were implemented but were limited to extraction of GS from pathology notes alone [6,7]. However, in the real world, pathology notes are often unavailable for a proportion of patients, especially patients who switch hospital systems during the course of their oncologic care, which happens more frequently for prostate cancer patients due to their longer survival times. Therefore, for many patients, the only source of GS is from their clinical notes. One previous single-institution project also recognized the importance of extraction from clinical notes and sought to extract GS from both clinical and pathology notes [8]. However, they were only able to extract GS with an accuracy of 91% and only from prostate surgery GS. By comparison, our NLP system had an accuracy 95% accuracy, which compares favorably, and we were able to extract both prostate surgery and biopsy GS. To our knowledge, no previously published systems were able to extract both prostate surgery and prostate biopsy GS from clinical notes.

Our NLP system has many important strengths. First, this NLP system was designed from the ground up by a practicing oncologist. Thus, the NLP system was organized and built from the start to best mirror the workflow and thought processes of an oncologist reading medical notes, taking advantage of the various mental shortcuts used by domain experts. Second, we developed our NLP system to extract from both pathology and clinical notes. Pathology notes are generally more structured and thus an easier task for NLP systems, which is why most previous GS extraction systems only worked on pathology notes. On the other hand, clinical notes are much less structured, and thus applying NLP systems to clinical notes with great accuracy is a harder task. Third, we required that our NLP system extract GS from both prostate surgery and prostate biopsies, something which has not been accomplished before. This task is trivial for pathology notes (as pathology notes are usually clearly labeled as either prostate surgery or prostate biopsy) but is much more difficult for clinical notes where this information needs to be gleaned from the free text and placed into context. Finally, we were able to design and successfully implement an NLP-assisted extraction system that achieved significantly higher accuracy rates than did the NLP-only extraction system. It should be noted that our algorithm for distinguishing between uncomplicated and complicated notes played a pivotal role in the high accuracy of our NLP system, as the NLP-human synergy can only work if the proper notes are assigned to human extractors. Here, we again took advantage of the expert domain knowledge available to us and leveraged a clinically based algorithm.

Our study does have some notable limitations. First, we selected only 200 patients for manual review, and therefore the study was not powered to detect small differences in accuracy between the different extraction methods. However, even with only 200 patients reviewed, we were still able to detect a statistically significant difference in accuracy between the NLP-assisted and NLP-only extraction models. Second, we conducted a single-institution study, and therefore external validity may be limited. However, our institution notably does not have standardized templates for clinical notes between health care providers. Thus, our clinical notes likely contained a wide variability of wordings, sentence structures, and note formatting between different providers, similar to those of other institutions.

In conclusion, we successfully designed and implemented an NLP-assisted extraction system to extract Gleason scores from medical notes with almost 99% accuracy, a significantly faster and cheaper solution over human extraction alone. In future works, we will expand our NLP-assisted extraction system for the extraction of other clinically important variables.

## Appendix

Multimedia Appendix 1 Pseudocode for the natural language processing modules.

Multimedia Appendix 2 Characterization of natural language processing errors among uncomplicated notes.

Multimedia Appendix 3 Breakdown of accuracy for human and natural language processing extraction between prostate surgeries and biopsies.

## Abbreviations

GS: Gleason score
EHR: electronic health record
ICD: International Classification of Disease:
NLP: natural language processing
P: primary Gleason score
S: secondary Gleason score
T: total Gleason score
