# Supplementary material for: A Natural Language Processing–Assisted Extraction System for Gleason Scores: Development and Usability Study
Source: JMIR Cancer. 2021 Jul 2;7(3):e27970. doi: 10.2196/27970 (PMC8285739; doi:10.2196/27970)
Supplement: Multimedia Appendix 1 [file cancer_v7i3e27970_app1.docx]

**Supplemental Table 1:** Pseudocode for the NLP modules

**A:** Modules for pathology notes

| **A1: Classifier module** for pathology notes |
| --- |
| **For** each pathology note: |
| **Step 1:** Remove all names from note.  *This step was important because some patients can have last names which overlapped with relevant keywords*  *Ex: “John Gleason”* |
| **Step 2:** Remove all clinical information from note.  *This step was important because clinical information sections of pathology reports may contain keywords which describe*  *earlier procedures, rather than the current procedure*  *Ex: “Clinical Information: 67-year-old male with history of prostate cancer diagnosed on biopsy, presents for RRP”* |
| **Step 3:** Search note for presence of:  [surgery keywords] - *Ex: “Prostatectomy”, “RRP”, “RALP”*  [cancer positive keywords] - *Ex: “Gleason”* |
| **Step 4:** Report classifications:  If [cancer positive keywords] found and [surgery keywords] found, then pathology note = prostate surgery.  If [cancer positive keywords] found and [surgery keywords] not found, then pathology note = prostate biopsy. |
| **A2: Extractor module** for prostate surgery pathology notes |
| **For** each prostate surgery pathology note: |
| **Step 1:** Extract primary (P), secondary (S), and total (T) GS components:  **Step 1A:** Search note for scores in any of the following formats: “P+S” or “P+S=T” or “T=P+S” or “P+S(T)” or “(T)P+S”  **Step 1B:** If no scores were found, search note for presence of:  [primary keywords] - *Ex: “Primary Gleason”, “Primary Pattern”, “Grade”, etc.*  [secondary keywords] - *Ex: “Secondary Gleason”, “Secondary Pattern”, etc.*  [tertiary keywords] - *Ex: “Tertiary”, etc.*  [total keywords] - *Ex: “Gleason”, “Score”, etc.*  Then set GS components:  If [primary keywords] found and [tertiary keywords] not found nearby, then P = number following [primary keyword]  If [secondary keywords] found and [tertiary keywords] not found nearby, then S = number following [secondary keyword]  If [total keywords] found and [primary keywords] not found and [secondary keywords] not found and [tertiary  keywords] not found nearby, then T = number following [total keywords]  **Step 1C:** If only 2 out of 3 GS components were found, calculate last component based on equation P+S=T |
| **A3: Extractor module** for prostate biopsy pathology notes |
| **For** each prostate biopsy pathology note: |
| **Step 1:** Extract primary (P), secondary (S), and total (T) GS components (same as algorithm above) for each mention of GS |
| **Step 2:** Calculate maximum GS if prostate biopsy pathology note has multiple GS |

**B:** Modules for clinical notes

| **B1: Extractor module** for clinical notes |
| --- |
| **For** each clinical note: |
| **Step 1:** Extract primary (P), secondary (S), and total (T) GS components (same as algorithm above) for each mention of GS |
| **Step 2:** Classify GS as prostate biopsy or prostate surgery using classifier module (see below) for each mention of GS |
| **Step 3:** Calculate maximum GS among all GS classified as prostate surgery |
| **Step 4:** Calculate maximum GS among all GS classified as prostate biopsy |
| **B2: Classifier module** for clinical notes |
| **For** each mention of GS: |
| **Step 1:** Search the immediate sentence surrounding GS mention for presence of:  [more specific surgery keywords] - *Ex: “Prostatectomy”, “RRP”, “RALP”, etc.*  [less specific surgery keywords] - *Ex: “Biopsy”, “Bx”, “Core”, etc.*  [more specific biopsy keywords] - *Ex: “Pathologic Stage”, “pT2”, “pT3”, etc.*  [less specific biopsy keywords] - *Ex: “Clinical Stage”, “cT1c”, etc.* |
| **Step 2:** Calculate classification:  If [more specific surgery keywords] found and [more specific biopsy keywords] not found, then classification = surgery  If [more specific surgery keywords] not found and [more specific biopsy keywords] found, then classification = biopsy  If [more specific surgery keywords] found and [more specific biopsy keywords] found, then classification = unknown  If [more specific surgery keywords] not found and [more specific biopsy keywords] not found, then classification = unknown |
| **Step 3:** If classification is still unknown, then:  If [less specific surgery keywords] found and [less specific biopsy keywords] not found, then classification = surgery  If [less specific surgery keywords] not found and [less specific biopsy keywords] found, then classification = biopsy  If [less specific surgery keywords] found and [less specific biopsy keywords] found, then classification = unknown  If [less specific surgery keywords] not found and [less specific biopsy keywords] not found, then classification = unknown |
| **Step 4:** If classification is still unknown, then search the three sentences surrounding GS mention for presence of keywords used in Step 1. |
| **Step 5:** Calculate classification, based on same algorithm as Step 2. |
| **Step 6:** If classification is still unknown, then calculate classification, based on same algorithm as Step 3. If classification is still unknown, then disregard GS. |

**C:** Modules for GS aggregator

| **GS aggregator module** |
| --- |
| For **each patient**: |
| **Step 1A:** Calculate maximum GS for prostate surgery pathology notes if patient has multiple prostate surgery pathology GS |
| **Step 1B:** Calculate maximum GS for prostate biopsy pathology notes if patient has multiple prostate biopsy pathology GS |
| **Step 1C:** Calculate maximum GS for prostate surgery clinical notes if patient has multiple prostate surgery clinical GS |
| **Step 1D:** Calculate maximum GS for prostate biopsy clinical notes if patient has multiple prostate biopsy clinical GS |
| **Step 2:** Flag GS as complicated if only P component found, only S component found, or P+S≠T |
| **Step 3:** Calculate prostate surgery GS:  If only prostate surgery pathology GS was found, then GS = prostate surgery pathology GS  If only prostate surgery clinical GS was found, then GS = prostate surgery clinical GS  If both prostate surgery GS were found and the same, then GS = common prostate surgery GS  If both prostate surgery GS were found and different, then flag GS as complicated |
| **Step 4:** Calculate prostate biopsy GS:  If only prostate biopsy pathology GS was found, then GS = prostate biopsy pathology GS  If only prostate biopsy clinical GS was found, then GS = prostate biopsy clinical GS  If both prostate biopsy GS were found and the same, then GS = common prostate biopsy GS  If both prostate biopsy GS were found and different, then flag GS as complicated |
| **Step 5:** Flag GS as complicated if prostate surgery GS ≠ prostate biopsy GS |

GS = Gleason score. Comments are displayed in *italics*. Keyword arrays are denoted by [square brackets].
