# Supplementary material for: A Natural Language Processing–Assisted Extraction System for Gleason Scores: Development and Usability Study
Source: JMIR Cancer. 2021 Jul 2;7(3):e27970. doi: 10.2196/27970 (PMC8285739; doi:10.2196/27970)
Supplement: Multimedia Appendix 2 [file cancer_v7i3e27970_app2.docx]

**Supplemental Table 2:** Characterization of NLP errors among uncomplicated notes

| Category | Count | Example |
| --- | --- | --- |
| Data entry mistake in the original note | 1 | “Gleason score 4+4, Primary Pattern Grade 4, Secondary Pattern Grade 3” was interpreted as Gleason 4+4, but note later states the 4+4 was an error in data entry |
| Sentence complexity | 2 | “Prostate Bx at outside hospital with Gleason 8, plan prostatectomy with surgeon today” was incorrectly interpreted as prostatectomy Gleason due to proximity to the word prostatectomy |
| Hypothetical situations in the note | 1 | “I discussed performing a prostatectomy for this patient with Gleason 7 disease” was incorrectly interpreted as if the prostatectomy was positive for Gleason 7 disease |
| Miscellaneous reason | 1 | “S/p rrp Gleason VI” was not picked up by the NLP system because it was programmed to pick up only alphanumeric numbers |
