# Supplementary material for: A Natural Language Processing–Assisted Extraction System for Gleason Scores: Development and Usability Study
Source: JMIR Cancer. 2021 Jul 2;7(3):e27970. doi: 10.2196/27970 (PMC8285739; doi:10.2196/27970)
Supplement: Multimedia Appendix 3 [file cancer_v7i3e27970_app3.docx]

**Supplemental Table 3:** Breakdown of accuracy for human and NLP extraction, between prostate surgeries and biopsies

|  | Prostate Surgery | Prostate Biopsy | Total |
| --- | --- | --- | --- |
| Uncomplicated Notes (no.) | 193 | 188 | 381 |
| Human Error (no.) | 4 | 6 | 10 |
| Human Accuracy (%) | 97.9% | 96.8% | 97.4% |
| NLP Error (no.) | 5 | 0 | 5 |
| NLP Accuracy (%) | 97.4% | 100.0% | 98.7% |
| Complicated Notes (no.) | 7 | 12 | 19 |
| Human Error (no.) | 0 | 0 | 0 |
| Human Accuracy (%) | 100.0% | 100.0% | 100.0% |
| NLP Error (no.) | 5 | 9 | 14 |
| NLP Accuracy (%) | 28.6% | 25.0% | 26.8% |
| Total | 200 | 200 | 400 |
